# Parents’ and Children’s Acceptance of Silver Diamine Fluoride Application on Primary Teeth in the United Arab Emirates

**DOI:** 10.3290/j.ohpd.b3680331

**Published:** 2022-12-13

**Authors:** Tarun Walia, Raghavendra M. Shetty, Lara M. Al-Sammarraie

**Affiliations:** a Associate Professor, Unit of Paediatric Dentistry, Department of Clinical Sciences, College of Dentistry, Ajman University, Ajman, UAE. Study concept and design, data interpretation, drafted the manuscript.; b Assistant Professor, Department of Clinical Sciences, College of Dentistry, Ajman University, Ajman, United Arab Emirates. Drafted and revised the manuscript, approved the final version.; c Postgraduate Student, Master of Science – Paediatric Dentistry, College of Dentistry, Ajman University, Ajman, United Arab Emirates. Data acquisition, analysis, and interpretation, drafted the manuscritp in partial fulﬁlment of requirements for a degree.

**Keywords:** children, parent preferences, primary teeth, silver diamine fluoride, United Arab Emirates

## Abstract

**Purpose:**

To assess the acceptance of silver diamine fluoride (SDF) application on children’s teeth among parents and children in the United Arab Emirates (UAE).

**Materials and Methods:**

A pre-tested questionnaire and clinical photographs of SDF-treated teeth were employed to determine preferences among 370 parents for its use in managing dental caries in their children’s teeth. A similar number of children ages 4 to 8 years were also interviewed, and their reaction to SDF was assessed through a facial image scale after showing pictures of pre- and post-SDF treated primary teeth.

**Results:**

265 mothers and 105 fathers participated in this study. The Χ^2^ test was used to test for the statistically significant differences between parental perceptions. Almost all parents responded that SDF was either unacceptable or extremely unacceptable for their children’s anterior teeth in comparison to 63% for posterior teeth (p = 0.009). Fathers were more comfortable with SDF treatment for posterior teeth on a scale of 3.1 out of 4, in comparison to 1.8 for mothers (p = 0.007). Parents with limited education (up to primary school) showed greater SDF acceptance in comparison to college-graduate parents (p = 0.000). The level of parental SDF acceptance increased with the children’s behavioural barriers. The older children had a lower acceptance rate, at 1.2 and 2.5 for anterior and posterior teeth, respectively (p = 0.000).

**Conclusions:**

SDF was acceptable to UAE-based parents for posterior teeth; however, they preferred it for anterior teeth only when advanced behavioural management (e.g., sedation) was advocated. Socioeconomic factors moderated their SDF preference regarding the location of tooth and treatment difficulty. Younger children were relatively more receptive to SDF use than were older children.

The topical application of silver diamine fluoride (SDF) has been advocated as a “non-invasive caries progression inhibition method” to manage active lesions, particularly when conventional operative strategies cannot be applied in children with limited cooperative behaviour. If the required treatment is not provided in time, it will eventually lead to odontogenic infections resulting in endodontic treatment or premature loss of teeth due to extraction.^10^ In such situations, advanced pharmacological behaviour management methods, such as sedation or general anesthesia, are often warranted to provide high-quality dental treatment. However, many parents do not prefer these techniques, not only because they are more expensive.^20^

Dental caries in the United Arab Emirates (UAE) is still a major paediatric health problem, as concluded in a systematic review conducted by Al Anouti et al.^2^ The prevalence rate in 5-year-old children in the UAE is about 74%.^15^ The use of SDF has been drawing increasing attention among dental practitioners.^18^ It has an anti-bacterial effect, enhances remineralisation of dentin, arrests caries, and can replace stressful restorative procedures in young children.^10^ In UAE, the authorities too have allowed its use as a part of minimal intervention caries management since the outbreak of the COVID-19 pandemic.^13^

However, SDF causes black discolouration of carious enamel and dentin, which may be an obstacle to its usage. Parents may refuse to accept its topical application, as the staining affects the child’s aesthetic appearance, particularly if applied to anterior teeth. The literature shows that parental apprehension about the tooth discolouration associated with SDF is a concern for many providers.^9^ Up to now, no data has been available from the UAE on parental preferences for SDF application on children’s teeth. Moreover, aesthetic concerns in primary teeth have been studied mainly from the point of view of parents. The children’s aesthetic perception should also be given due consideration while planning a study, as they are conscious of their own dental aesthetic appearance and that of the other children.^8^ To the best of the present authors’ knowledge, no such study has investigated the post-SDF aesthetic concerns in children. The purpose of this quantitative study was to evaluate SDF acceptance among UAE-based parents in the management of their children’s caries in different clinical scenarios, and also to understand the reaction of their children toward SDF application. The null hypothesis tested was that there is no difference between parental and child acceptance levels of SDF treatment.

## Materials and Methods

Ethical approval was obtained from the institutional research ethics committee.

### Participants

Three hundred seventy parents who attended dental clinics for treatment of their children’s teeth and fulfilled the established inclusion requirements were selected. The sample size was calculated using statistical power analysis software (G Power version 3.1.9.7), considering a margin of error of 0.5% with a power of 80%. The participation criteria were parents who have had at least one child with a previous dental experience that included caries removal by rotary handpiece, the age of the child was between 4 to 8 years old, and agreement to participate was given by signing the consent form. If the participating parents had more than one child who had prior experience with rotary dental instruments, they were asked to respond about their youngest child. Parents experiencing their child’s first dental visit or those with a child that did not have any previous dental treatment experience with handpiece were excluded.

### Questionnaire

A previously developed and tested parental questionnaire was modified according to the requirements of the present study.^7^ The revised version was further pre-tested for both face and content validity to avoid two-in-one, confusing, and leading questions. The questionnaire survey was pilot tested on a subset of 20 parents and children who were not a part of the final sample. Five faculty members were given the same questionnaire and asked individually to assess its content validity and rate each question using a 4-point scale. The revised questionnaire was also distributed to a representative sample, and survey administration was then repeated after two weeks amongst the same group to assess its consistency and reliability.

The main investigator was then trained and calibrated in the method of filling out the questionnaire through a pilot study on a set of 10 parents with their children. They were given detailed explanations about the questionnaire and its coding manual and rubrics. Initially, structured information on application steps, cost, advantages, and disadvantages of SDF was delivered to the parents. The details given to them included: SDF is a liquid that can be painted on the tooth cavity, and this solution will stop the cavity from becoming larger. Parents were also informed that the liquid changes the cavity colour, which will make it darker. Reassurance was given to them that the darker colour serves as a guide that ensures the treatment is effective. Parents then observed high-quality colour clinical photographs of dental caries in anterior as well as posterior primary teeth before and after SDF application. They were asked if the post-treatment discolouration shown through the photographs would be acceptable to them. Their queries were answered in a standardised manner and responses were filled in on a Google form. After the parents finished filling out the questionnaire, the investigator met the child and explained that the SDF application is a simple, quick method, and does not require the use of a handpiece. There would be no shrill noise; furthermore, akin to nail polish on nails, it is a polish for the teeth. All the children were given similar information irrespective of their age group to maintain uniformity.

The first section of the questionnaire had information on demographic data of the participants, including their socioeconomic and educational status, ethnicity, number of children, and age of their child. In the second part, parents were asked about their child’s cooperation during the previous dental experience. In the third section of the questionnaire, parents were given five different situations where their child needed a dental filling, to determine their preference for SDF in that particular situation. Scenario 1 evaluated parental SDF acceptance levels even if their child was cooperative enough to do the filling; scenario 2 checked their preference for SDF if the child was upset, but could cooperate enough to complete the fillings. The third scenario was their acceptance levels where the child kicked or screamed and was not cooperative enough to complete the restoration; while the fourth and fifth situations assessed whether parents favoured the use of SDF in place of either nasal sedation or general anesthesia to perform the fillings. Each of the five scenarios was further divided into primary anterior and posterior teeth. To rate the acceptance of SDF treatment, parents were given choices on a 4-point Likert scale ranging from extremely unlikely, slightly unlikely, slightly likely, to very likely. The last part of the questionnaire had a facial image scale (FIS) for the children to assess their own reactions if SDF were applied to their teeth.

### Interview

The questionnaires were completed through a personal meeting. If both parents were accompanying the child on the day of the interview, then the preference was given to the mother to participate in the questionnaire completion. Special efforts were made to ensure that the parent understood each question and that their queries were answered before they selected an option. Similarly, children were given information about SDF in simple language and they selected a face from the FIS that reflected their feelings after they had a look at associated post-treatment discolouration through clinical photographs. After every ten questionnaires, parent and child-completed entries on the data collection sheet were re-checked to ensure there were no unanswered questions.

### Statistical Analysis

The collected data were tabulated and statistically analysed using Windows SPSS, version 27 (IBM; Chicago, IL, USA). Frequency analysis was carried out to obtain the percentage of the responses, while the Χ^2^ test was used to determine statistically significant differences between the responses and each independent variable. The dependent variable was the parental acceptance of staining associated with SDF treatment, and the independent variables were parental gender, education level, age of the child, and family income. Statistical significance was set at 5% (p < 0.05).

## Results

A total of 370 parents (265 mothers and 105 fathers) participated in the current study. The greatest number of parents (64%) were 31–40 years old (Table 1). Sixty-one percent of children were 6–7 years old, while 20% of the remaining children were 4–5 years old and 8 years old. The majority of parents (78%) were of Arab nationality, followed by 15% of South Asian descent, with a few Gulf Cooperation Council nationals and Filipinos. Parental education status varied from 65% who completed middle or high school, to 34% with a college degree. In terms of monthly income, the highest number of respondents (64%) was in the 5000–10,000 AED income group, and around 35% of parents had a combined family income of 10,000–15,000 AED.

**Table 1:** Demographic characteristics of parents responding to the survey (n = 370)

Variables	n (%)
**Gender**	
Female	265 (71.7)
Male	105 (28.3)
**Age (years)**	
≤30	20 (5.4)
31–40	237 (64.1)
41–50	106 (28.6)
≥51	7 (1.9)
**Education**	
Primary school	4 (1.1)
Middle school	74 (20.0)
High school	167 (45.1)
Graduated from college	125 (33.8)
**Income (per month)**	
0 AED – 5000 AED*	1 (0.3)
5000 AED – 10,000 AED	236 (63.8)
10,000 AED – 15,000 AED	129 (34.9)
15,000 AED – 20,000 AED	4 (0.5)
**Ethnicity**	
Arab	287 (77.6)
Filipino	13 (3.5)
Gulf Council Countries (GCC)**	15 (4.1)
South Asian	55 (14.9)
**Child’s age (years)**	
4–5	71 (19.2)
6–7	226 (61.1)
8	73 (19.7)

*AED: Arab Emirati Dirhams. **GCC: Alliance of Saudi Arabia, Kuwait, United Arab Emirates, Qatar, Bahrain, and Oman.

**Fig 1:**
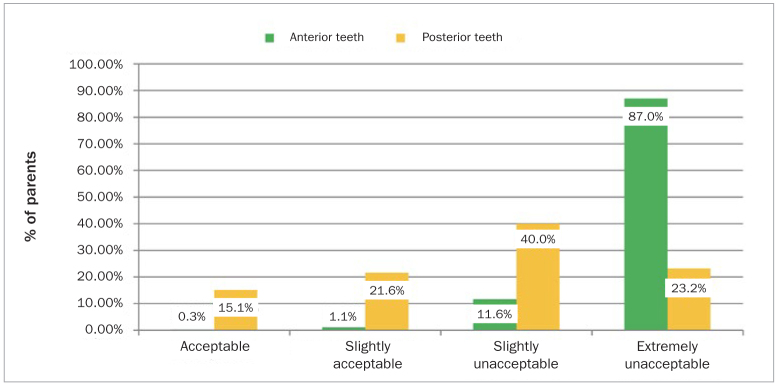
Parental acceptance of SDF based on aesthetics (p = 0.009).

**Fig 2:**
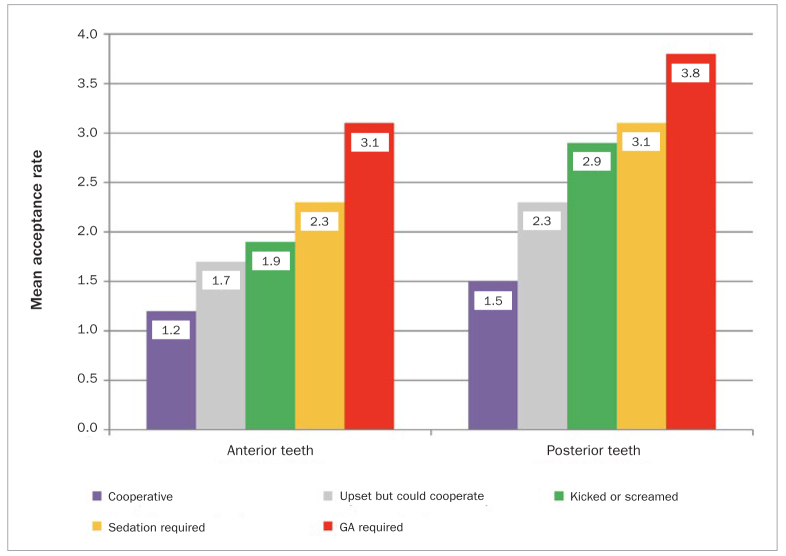
Parents’ mean acceptance scores of SDF treatment based on the child’s behaviour and tooth location (p = 0.002).

Almost all parents responded that SDF was either unacceptable or extremely unacceptable for anterior teeth, decreasing to 63% for posterior teeth (Fig 1). This distribution was statistically significantly different (p < 0.009). SDF preference levels of UAE-based parents increased with their children’s increased difficulty in receving a conventional filling in both anterior and posterior teeth. The results are presented in terms of their mean acceptance rating scores, ranging from 1 to 4 points, with 1 indicating “extremely unlikely” and 4 indicating “very likely” (Fig 2). It was found that the mean parental score was low for both anterior and posterior segments of teeth if the child was cooperative enough to receive a conventional restoration with 1.2 and 1.5 points for anterior and posterior teeth, respectively. However, the average parental acceptance levels increased to 3.1 points for the anterior teeth and 3.8 points for the posterior teeth in extreme situations, where their child would require pharmacological intervention such as general anesthesia to receive a dental restoration. This difference was statistically significant (p = 0.002).

Parental mean acceptance levels of SDF by gender were quite similar in terms of anterior teeth (Table 2). However, the difference was more noticeable for posterior teeth, with fathers’ acceptance of SDF being twice that of the mothers’ for posterior teeth, that is, the fathers’ score was 3.1 vs 1.8 for mothers. This difference was also statistically significant (p = 0.007). It is also apparent from Table 2 that parents became more receptive to SDF for both anterior and posterior teeth with an increase in their age. Mean acceptance levels of parents in the age group of ≤30 years had scores of 1.3 and 1.8 for anterior and for posterior teeth, respectively. The corresponding scores for parents who were in the older age group ≥40 years had a statistically significantly higher acceptance level of SDF compared to the younger group, with a score of 2.3 for anterior teeth and 3.2 for posterior teeth (p = 0.003).

**Table 2:** Parental preferences according to their demographic characteristics and children’s reaction to SDF treatment according to age

Parental demographic variable*	Mean acceptance rating**		p-value*
	Anterior teeth	Posterior teeth	
**Gender**			
Female (mother)	1.1	1.3	0.007*
**Age (years)**			
≤30	1.3	1.8	0.003*
31 – 40	1.7	2.5	
41 – 50 ≥51	2.3	3.2	
	2.5	3.6	
**Education**			
Primary school	2.4	2.7	0.000*
Middle school	2.1	2.5	
High school	1.5	2.1	
College graduate	1.1	1.3	
**Income**			
0 – 5000 AED	2.8	3.9	0.005*
5000 – 10,000 AED	2.3	3.1	
10,000 – 15,000	1.8	2.6	
15,000 – 20,000 AED	1.1	2.3	
**Children’s age (years)**			
4–5	2.3	3.1	0.000*
6–7	1.8	2.7	
8	1.2	2.3	

**Rating scale: 1: extremely unlikely; 2: slightly unlikely; 3: slightly likely; 4 very likely. *Statistically significant.

Similar trends were observed when parental preferences were compared with their education and income (Table 2). Parents in lower educational brackets and lower-income groups were more receptive to SDF for their children’s teeth. Parents who were educated only until primary school had a higher acceptance rate at 2.4 for anterior teeth and 2.7 for posterior teeth, in comparison to participant parents with a higher level of education. Those who finished high school or college had a lower acceptance score of up to 1.5 for anterior and 2.1 for posterior teeth. It was also seen that the mean acceptance rating was highest at 2.8 and 3.9 for anterior and posterior teeth, respectively, in the lowest parental income group of 0–5000 AED per month. Parents who had the highest income of 15,000–20,000 AED per month showed the least acceptance of SDF; this was statistically significant (p = 0.005).

It was quite evident that older children had the least preference for SDF for both anterior and posterior teeth (Table 2). Children 4–5 years old had a mean acceptance score of 2.3 and 3.1 for anterior and posterior teeth, respectively, while the corresponding rates for children 6–7 years old were 1.8 and 2.7. The 8-year-olds had the lowest acceptance score for anterior teeth – 1.2 – vs 2.5 for posterior teeth; this difference was statistically significant (p = 0.000).

## Discussion

Contrasting results have been obtained from various studies conducted in different parts of the world to assess parental perceptions of SDF staining. In a study^7^ conducted in the New York area of the US, parental acceptance levels were 75%, while another study from a community dental clinic in Oregon, USA, measured parental acceptance of SDF at 83%.^10^ More than 90% of US-based program directors in paediatric dentistry believe that parental acceptance of SDF is a concern.^16^ In two studies published in Saudi Arabia, the majority of 222 parents rejected this kind of treatment,^3^ while in another study 43% of parents objected to the staining caused by SDF treatment.^5^ However, in a clinical trial carried out in India with 38% SDF to arrest active carious lesions, 91% of participating parents approved the treatment.^20^ A survey done in China concluded that the easy and pain-free application of SDF compared to the conventional ‘drill-and-fill’ approach was considered advantageous by parents.^23^

Evidence obtained from the above-mentioned studies implies that parental concerns about the SDF staining tend to vary. The fact should be acknowledged that these surveys took place in settings where aesthetic concerns are different, and therefore cannot be compared with standards of other countries.^5^ Aesthetic considerations are important for parents in the selection of their children’s dental treatment. Holan et al^13^ found that parents always preferred to have an aesthetic treatment for anterior teeth for their children. The current study is the only investigation from the UAE which has evaluated children’s reaction to SDF dark discolouration on their primary anterior and posterior teeth along with the respective parental preferences.

One of the salient findings obtained from the current survey is the higher parental acceptance of SDF for posterior teeth than in the anterior teeth in all situations, which is similar to the results of many other studies.^3,5,7^ This is probably because the parents were conscious of their children’s dental aesthetics and were sure that the posterior teeth would not show when smiling and hence not affect their children psychologically. Many parents agreed to SDF treatment to avoid sedation or general anesthesia as a treatment option in the present study. This outcome also resembles the surveys carried out by Akshitha et al^4^ and Crystal et al.^7^ The data suggest that parents try to avoid pharmacological behavioural management to deliver the required treatment for their children’s carious teeth. This also can be due to parental opinions on pharmacological methods being more hazardous for their child’s life as well as costlier vs SDF treatment.

In this study, both mothers and fathers had a lower acceptance level for SDF treatment when indicated for anterior vs posterior teeth. This is in contrast with a survey done by Clemens et al,^6^ who inferred that most parents agreed or strongly agreed about their discomfort with post-SDF application staining. The finding from the present study that fathers highly accepted SDF for posterior teeth in comparison to mothers was also different from the results of a study which found that both genders had similar low acceptance of SDF treatment in posterior teeth due to aesthetics.^3^ Peretz and Ram^17^ concluded that older parents preferred to have a quicker dental procedure for their children. This was the case in the present study as well, where parental preferences were highly influenced by their age. The younger the parents, the lower their acceptance of SDF treatment; this preference increased for both anterior and posterior teeth with an increase in parents’ age. More highly educated parents were more concerned about the aesthetic appearance of their children’s teeth.^1^ This agrees with the results of the present survey, where the parents who were educated up to college or high school had a lower acceptance level of SDF treatment.

The SDF acceptance levels were higher among 4- to 5-year-olds; as expected, their preference levels decreased as they grew older. Their responses may also have been influenced by the fact that they found the noise and vibration of the handpiece annoying, and hence preferred SDF. A study assessed the effect of noises made by dental instruments on dental fear in children and found that children aged 6 years or younger were most frightened of the handpiece noise.^15^ The children’s reaction to SDF was assessed in this study through a facial image scale (FIS), as very young children lack the cognitive ability to complete questionnaires, and indirect behavioural measurements are the only real alternative to evaluate their preferences.^19^ The FIS contains some pictures that are ambiguous in what they portray and children took time to answer. However, a systematic review and meta-analysis on the effectiveness of various children’s dental anxiety scales used in paediatric dentistry has concluded that the FIS scale is equivalent to other methods in assessing anxiety levels in a paediatric population.^22^

This study can have a positive effect on the management of caries, as parental acceptance is a major factor in choosing SDF and lack of knowledge could be a barrier to the acceptance of SDF. Well-designed randomised controlled trials should be planned to assess parental acceptance of SDF in primary and permanent teeth, especially for children with special healthcare needs and in low socioeconomic-status communities. Decision-making factors such as cost, safety, and alternatives for more aesthetic and permanent restorations as their child’s cooperation improves should also be evaluated to study the parental preferences for SDF treatment.

### Limitations

Parental responses could have been influenced by perceived pressure to respond positively or as a response to the success of SDF treatment. The results obtained would have been more accurate if the questionnaire had been filled in after the clinical application of the SDF on their children’s teeth and not by showing pre- and post-SDF application pictures. None of the parents who participated had a child who had been treated with SDF. This study included very few high-income parents, which could also influence the results. Children self-reported their SDF preferences with a facial image scale. This could have led to some misinterpretation, as it can be difficult for them to discriminate well between drawings of facial expressions.

## Conclusion

UAE-based parents were more receptive when SDF was indicated for posterior than anterior teeth. However, their preference increased when the provision of dental treatment required behavioural management techniques such as sedation or general anesthesia. Parents in the older age group as well as with lower educational and income status had higher SDF acceptance levels. Younger children were more willing to have SDF applied to their teeth than were older children.

## References

[ref1] Al-Batayneh OB, Al-Khateeb HO, Ibrahim WM, Khader YS (2019). Parental knowledge and acceptance of different treatment options for primary teeth provided by dental practitioners. Front Public Health.

[ref2] Al Anouti F, Abboud M, Papandreou D, Haidar S, Mahboub N, Rizk R (2021). Oral health of children and adolescents in the United Arab Emirates: a systematic review of the past decade. Front Oral Health.

[ref3] Al shammari AF, Almuqrin AA, Aldakhil AM, Alshammari BH, Lopez JNJ (2019). Parental perceptions and acceptance of silver diamine fluoride treatment in Kingdom of Saudi Arabia. Int J Health Sci.

[ref4] Akshitha E, Girish SR, Shankar S, Lalithambigai G, John S (2022). Assessment of parental perceptions and acceptance of silver diamine fluoride staining among the children with dental caries experience in Namakkal, Tamil Nadu – A cross-sectional study. J Global Oral Health.

[ref5] Bagher SM, Sabbagh HJ, AlJohani SM, Alharbi G, Aldajani M, Elkhodary H (2019). Parental acceptance of the utilization of silver diamine fluoride on their child’s primary and permanent teeth. Patient Prefer Adherence.

[ref6] Clemens J, Gold J, Chaffin J (2018). Effect and acceptance of silver diamine fluoride treatment on dental caries in primary teeth. J Public Health Dent.

[ref7] Crystal YO, Janal MN, Hamilton DS, Niederman R (2017). Parental perceptions and acceptance of silver diamine fluoride staining. J Am Dent Assoc.

[ref8] Di Blasio A, Mandelli G, Generali I, Gandolfini M (2009). Facial aesthetics and childhood. Eur J of Paediatr Dent.

[ref9] Elkhodary HM, Alaki SM, Bagher S (2015). Preferences of anterior and posterior dental restorative materials among children and parents. Dent J.

[ref10] Gao SS, Zhao IS, Hiraishi N, Duangthip D, Mei ML, Lo ECM (2016). Clinical trials of silver diamine fluoride in arresting caries among children: a systematic review. JDR Clin Trans Res.

[ref11] Grund K, Goddon I, Schüler IM, Lehmann T, Heinrich-Weltzien R (2015). Clinical consequences of untreated dental caries in German 5- and 8-year-olds. BMC Oral Health.

[ref12] Guidelines for the Provision of Dental Services during COVID-19 Version 2. Health Policies and Standards Department Health Regulation Sector (2021). Dubai Health Authority. https://www.dha.gov.ae/uploads/112021/8e4ad8ac-cd05-430b-b843-da811e214d67.pdf.

[ref13] Holan G, Rahme MA, Ram D (2019). Parents’ attitude toward their children’s appearance in the case of esthetic defects of the anterior primary teeth. J Clin Pediatr Dent.

[ref14] Kowash MB, Alkhabuli JO, Dafaalla SA, Shah A, Khamis AH (2017). Early childhood caries and associated risk factors among preschool children in Ras Al-Khaimah, United Arab Emirates. Eur Arch Paediatr Dent.

[ref15] Muppa R, Bhupatiraju P, Duddu M, Penumatsa NV, Dandempally A, Panthula P (2013). Comparison of anxiety levels associated with noise in the dental clinic among children of age group 6–15 years. Noise Health.

[ref16] Nelson T, Scott JM, Crystal YO, Berg JH, Milgrom P (2016). Silver diamine fluoride in pediatric dentistry training programs: survey of graduate program directors. Pediatr Dent.

[ref17] Peretz B, Ram D (2002). Restorative material for children’s teeth: preferences of parents and children. ASDC J Dent Child.

[ref18] Seifo N, Robertson M, MacLean J, Blain K, Grosse S, Milne R (2020). The use of silver diamine fluoride (SDF) in dental practice. Br Dent J.

[ref19] Shetty RM, Khandelwal M, Rath S (2015). RMS Pictorial Scale (RMS-PS): An innovative scale for the assessment of child’s dental anxiety. J Indian Soc Pedod Prev Dent.

[ref20] Shrivastava U, Barjatya K, Bharath BAK, Vatsal A, Shrivastava R, Manker A, Binti RC, Juneja P (2021). Effectiveness and parental perception of silver diamine fluoride toward treatment of dental caries in primary teeth. Int J Clin Pediatr Dent.

[ref21] Sivakumar P, Gurunathan D (2019). Behavior of children toward various dental procedures. Int J Clin Pediatr Dent.

[ref22] Tiwari S, Kulkarni P, Agrawal N, Mali S, Kale S, Jaiswal N (2021). Dental anxiety scales used in pediatric dentistry: a systematic review and meta-analysis. J Contemp Dent Pract.

[ref23] Zhi QH, Lo ECM, Lin HC (2012). Randomized clinical trial on effectiveness of silver diamine fluoride and glass ionomer in arresting dentine caries in preschool children. J Dent.

